# Comparison of Three Internal Fixation Constructs for AO/OTA 33-A3 Distal Femoral Fractures: A Biomechanical Study

**DOI:** 10.3390/bioengineering11111110

**Published:** 2024-11-04

**Authors:** Wei Xie, Hui Liu, Shufen Chen, Weizhen Xu, Weibin Lin, Tianlai Chen, Lingqi Zhu, Wenliang Zhai, Jin Wu

**Affiliations:** Department of Orthopaedics, The 909th Hospital, School of Medicine, Xiamen University, Zhangzhou 363000, China; xiewei12353@stu.xmu.edu.cn (W.X.); lhui175@163.com (H.L.); csf15280624490@163.com (S.C.); xwz0214@outlook.com (W.X.); m18350070607@163.com (W.L.); m15060024956@163.com (T.C.); lqzhu@stu.xmu.edu.cn (L.Z.); wlzhai1971@xmu.edu.cn (W.Z.)

**Keywords:** distal femoral fracture, internal fixation, intramedullary nail, plate, biomechanics

## Abstract

Background: To compare the biomechanical performance of three internal fixation constructs for AO/OTA 33-A3 distal femoral fractures. Methods: Thirty AO/OTA 33-A3 synthetic distal femoral fracture models were constructed and randomly divided into three groups. Group A (dual-plate construct) was fixed with a medial locking plate combined with a less invasive stabilization system (LISS). Group B was fixed with a retrograde femoral nail (RFN) combined with an LISS (RFN + LISS construct), and Group C was fixed with a retrograde tibial nail (RTN) combined with an LISS (RTN + LISS construct). The axial displacement, axial stiffness, torsional displacement, torsional stiffness and maximum failure load of different internal fixation constructs were recorded and statistically analyzed. Results: In the axial compression test, the average stiffness of Group C was significantly higher than that of Groups A and B, and the average displacement of Group C was significantly smaller than that of Groups A and B. In the torsion test, the torsion degree of Group C was significantly lower than that of Groups A and B, and Group C had a higher torsional stiffness than Groups A and B. In the axial compression failure test, the average ultimate load (a displacement greater than 5 mm) of Group C was significantly higher than that of Groups A and B. Conclusion: The biomechanical strength of the RTN combined with a plate is higher than that of the RFN combined with plate and dual-plate constructs, which can be used as an internal fixation option for the treatment of comminuted distal femoral fractures.

## 1. Introduction

Distal femoral fractures, which are defined as fractures occurring within 9 cm of the distal femur and involving the distal metaphysis or articular surface, are a prevalent type of injury in clinical cases. Specifically, they account for approximately 1% of all fractures and 3%–6% of femoral fractures. AO-A-type femoral fractures are particularly common among all femoral fracture types, highlighting their importance within distal femoral injuries [1,2]. Epidemiologically, the incidence of distal femoral fractures follows a bimodal distribution, with peaks in young men with high-energy injuries and older women with low-energy injuries [3,4]. Clinicians advocate surgical treatment for distal femoral fractures in order to achieve anatomic reduction, stable internal fixation and early physical exercise [5]. There are many complex distal femoral fractures, such as medial cortical defect, periprosthesis fracture, fracture nonunion, severe osteoporosis, comminuted distal femoral fracture, etc. If only a single plate was used to fix the femur, the fracture remained unstable due to the lack of mechanical support for the distal medial cortex. This can lead to the loss of reduction and the re-displacement of the fracture when patients perform knee functional exercises early after surgery, affecting the fracture healing [6,7,8,9,10,11]. Despite the fact that treatment failure of the femur fracture can be multifactorial, stable fixation was critical to provide an adequate healing environment to allow joint mobility and weight bearing early or immediately following treatment. At present, the possible complications of a single less invasive stabilization system (LISS) are often avoided by adding a medial locking plate or retrograde femoral nail (RFN) [12,13,14]. Locking plate internal fixation has angular stability, which can avoid the angular deformity caused by non-locking plate internal fixation. It also has a strong withdrawal force, which reduces the occurrence of internal fixation loosening, especially in patients with condylar fractures and osteoporosis fractures [15]. However, the medial plate may damage the bone blood at the fracture site and thus affect the healing of the fracture [16], and the RFN may affect the function of the knee joint due to the damage of cartilage at the point of insertion. Therefore, it is necessary to apply a new type of intensive fixation technique for these patients to reduce the occurrence of related complications. The retrograde tibial nail (RTN) is a new internal fixation type, often used in minimally invasive treatments of distal tibia fractures, and it has good biomechanical performance. Since an RTN is an intramedullary fixation, the soft tissue and blood flow of the medial fracture cortex are not damaged during installation. The insertion point of the RTN does not enter the knee joint and does not damage the cartilage and affect the knee joint function. However, whether the RTN combined with an LISS can achieve a good biomechanical RTN in the fixation of distal femoral fractures remains unclear. Therefore, we conducted biomechanical testing using artificial bones to compare the biomechanical efficacy of an RTN combined with an LISS versus an RFN combined with an LISS and dual-plate fixations for distal femoral fractures. We hypothesized that the RTN combined with an LISS fixation is superior to the RFN combined with an LISS and dual-plate fixations in this experiment.

## 2. Methods

### 2.1. Materials and Methods

**Figure 1:**
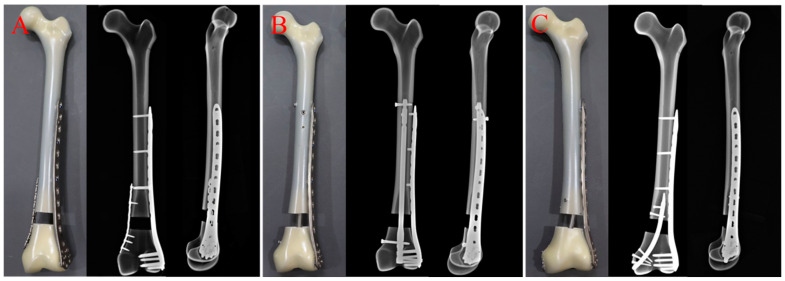
(**A**–**C**) Photographs and radiographic images that describe the fixation strategies of Group A (dual-plate construct), Group B (RFN + LISS construct), and Group C (RTN + LISS construct).

A comminuted supracondylar femur fracture (AO/OTA33-A3) gap model was created in thirty artificial femurs (fourth-generation synthetic composite bones; Type 3403; Pacific Research Laboratories, Vashon, WA, USA). A 2 cm gap osteotomy was performed with an oscillating saw at a distance of 6.5 cm from the proximal end of the intercondylar notch to simulate a distal supracondylar femoral extra-articular comminuted fracture after implantation [17]. All fracture models were randomly divided into three groups (Group A, Group B and Group C) on average (Figure 1A–C). Group A was a double-plate (dual-plate construct) group in which the fracture models were fixed with both internal and lateral plates. First, the distal lateral cortex of the model was fixed with a lateral plate (Double Medical, Xiamen, China; LPLC 04 Type I; 11 holes), 4.5 mm cortical screws were placed in holes 1, 4, and 7 at the proximal end of the plate, and 5.0 mm locking screws were placed in all of the holes at the distal end. The distal medial cortex of the model was fixed with a locking compression plate (Double Medical, Xiamen, China; LPSC 16 Type I; 9 holes), 3.5 mm cortical screws were placed in holes 1, 2 and 3 at the proximal end of the plate and holes 7, 8, and 9 at the distal end. Group B was a retrograde femoral nail combined with a lateral plate (RFN + LISS construct) group. First, the retrograde femoral intramedullary nail (Double Medical, Xiamen, China; LB-DFN-01; 9 × 280 mm) was placed in a standard operation, two screws were placed in the proximal end from the anterior–posterior direction and the left and right direction, respectively, and one screw was placed in the distal end from the inside to the outside. Then, the lateral plate was used for fixation, the distal end was also fixed with six screws, and the proximal end was fixed with three single cortical screws due to the obstruction of the nail. Group C was a retrograde tibial nail combined with a lateral plate (RTN + LISS construct) group. The lateral plate was first used to fix the fracture. The position, number and specifications of screws were the same as those of Group A. Then, the tibia was fixed by a retrograde tibial intramedullary nail (Double Medical, Xiamen, China; MA-TIN-01; 8 × 140 mm) and the entry point was selected about 0.5 cm in front of the medial epicondyle of the femur, with two screws placed in the proximal end and three screws placed in the distal end. The construction and operation of all models were carried out by the same formally trained senior chief physician in strict compliance with standardized surgical protocols and methodologies. After the osteotomies were completed, the distal end of each model was fixed to the operating table of the biomechanical machine by poly methyl methacrylate (PMMA). The results of the model were recorded under different stress conditions during the test.

### 2.2. Biomechanical Testing

**Figure 2:**
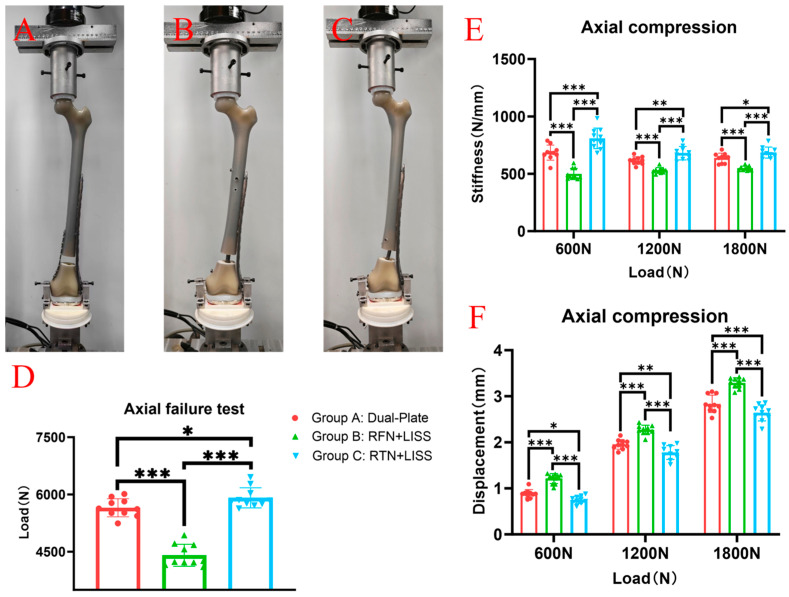
(**A**–**C**) The pictures of Group A (dual-plate construct), Group B (RFN + LISS construct) and Group C (RTN + LISS construct) in the axial compression test. (**D**) The results of the axial failure test showed that the ultimate load (displacement greater than 5 mm) of Group C was significantly higher than that of Group A and Group B. (**E**) The axial stiffness of Group C was significantly higher than that of Group A and Group B when the axial loads were 600 N, 1200 N and 1800 N. (**F**) The displacement of Group C was significantly smaller than that of Group A and Group B when the axial loads were 600 N, 1200 N and 1800 N. * *p* < 0.05, ** *p* < 0.01, *** *p* < 0.001.

**Figure 3:**
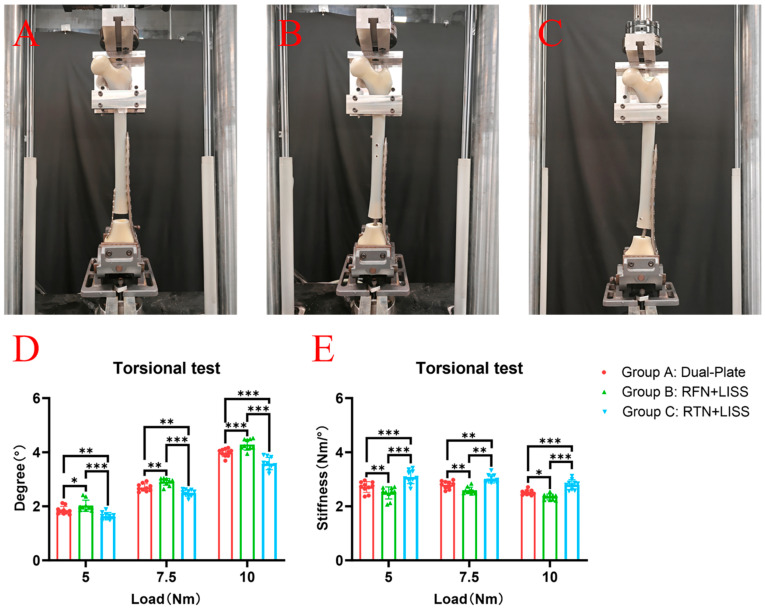
(**A**–**C**) The pictures of GroupA (dual-plate construct), Group B (RFN + LISS construct) and Group C (RTN + LISS construct) in the torsion test. (**D**) In the torsion test, the displacement degree of Group C was obviously smaller than that of Group A and Group B when the torque was 5 Nm, 7.5 Nm and 10 Nm. (**E**) In the torsion test, the torsional stiffness of the Group C was significantly greater than that of Group A and Group B when the torque was 5 Nm, 7.5 Nm and 10 Nm. * *p* < 0.05, ** *p* < 0.01, *** *p* < 0.001.

The same test location and equipment we have previously reported were used in this study; all experiments were performed at the Xiamen Medical Device Research and Development Testing Center using a mechanical testing machine (MTS Bionix Servo-hydraulics Test Systems Model 370.02; MTS Systems, Eden Prairie, MN, USA), and the same method was used to eliminate the specimen relaxation and creep before the formal test [18]. The model was fixed at an external rotation of 7° on the testing machine to ensure that the axial load passed through the mechanical axis of the femur, thereby simulating the force-bearing state of the femur in a normal adult during weight bearing. The axial compression load was gradually increased from 0 N to 1800 N at a speed of 50 mm/min, and data were recorded at 600 N, 1200 N and 1800 N (Figure 2A–C). Then, the femur was subjected to torsional loads from 0–10 Nm at a rate of 70 degrees/min, and the degrees were measured when the torque values were 5 Nm, 7.5 Nm and 10 Nm (Figure 3A–C). Finally, an axial compression load was applied with increments of 10 N/s until failure occurred, and the ultimate load was recorded. The results under the axial compressive load and torsional load were recorded, and the stiffnesses of the axial compression and torsion were calculated as the load (N) divided by the displacement (mm) (N/mm) and the torque (Nm) divided by the degree (Nm/°), respectively. In this study, the criteria for determining the stability failure of the fixation–bone–structure complex under stress were as follows: fracture, the loosening of the screws, or a displacement of the proximal fracture fragment greater than 5 mm.

### 2.3. Statistical Analysis

SPSS 24.0 software was used for the statistical analysis of the data. The axial compression displacement, axial compression stiffness, torsional displacement, torsional stiffness and ultimate failure load were analyzed by using the previous statistical methods. *p* < 0.05 was considered statistically significant.

## 3. Results

In this experiment, no screw loosening or fracture was observed in each group of specimens before the implementation of the destruction experiment.

In the axial compression test, the average stiffnesses of Group A, under the axial loads of 700 N, 1400 N and 1800 N, were 685 ± 66 N/mm, 616 ± 33 N/mm and 637 ± 42 N/mm, respectively. The average stiffnesses of Group B were 499 ± 48 N/mm, 529 ± 25 N/mm and 548 ± 19 N/mm, respectively. The average stiffnesses of Group C were 809 ± 90 N/mm, 680 ± 59 N/mm and 685 ± 48 N/mm, respectively. The average stiffness of Group C was significantly higher than that of Groups A and B (Figure 2E). The average displacements of Group A under axial loads of 700 N, 1400 N, and 1800 N were 0.88 ± 0.090 mm, 1.95 ± 0.105 mm and 2.84 ± 0.189 mm, respectively. The average stiffnesses of Group B were 1.21 ± 0.109 mm, 2.27 ± 0.102 mm and 3.29 ± 0.112 mm, respectively. The average stiffnesses of Group C were 0.75 ± 0.081 mm, 1.78 ± 0.150 mm, and 2.64 ± 0.176 mm, respectively. The average displacements of Group C were significantly smaller than that of Groups A and B (Figure 2F).

In the torsion test, the average degrees of Group A at torques of 5 Nm, 7.5 Nm and 10 Nm were 1.85 ± 0.144°, 2.69 ± 0.136° and 3.99 ± 0.144°, respectively. The average degrees of Group B were 2.02 ± 0.203°, 2.91 ± 0.127° and 4.28 ± 0.196°, respectively. The average degrees of Group C were 1.63 ± 0.134°, 2.49 ± 0.125° and 3.58 ± 0.228°, respectively. The torsion degrees of Group C were significantly lower than that of Groups A and B (Figure 3D). The average torsional stiffnesses of Group A are 2.71 ± 0.200 Nm/°, 2.79 ± 0.138 Nm/° and 2.51 ± 0.093 Nm/° at torques of 5 Nm, 7.5 Nm and 10 Nm, respectively. The average torsional stiffnesses of Group B are 2.49 ± 0.225 Nm/°, 2.59 ± 0.118 Nm/° and 2.34 ± 0.108 Nm/°, respectively. The average torsional stiffnesses of Group C are 3.08 ± 0.247 Nm/°, 3.01 ± 0.156 Nm/° and 2.80 ± 0.179 Nm/°, respectively. Group C had a higher torsional stiffness than Group A and Group B (Figure 3E).

In the axial compression failure test, the average limit loads (displacement greater than 5 mm) were 5652 ± 238 N for Group A, 4408 ± 289 N for Group B and 5913 ± 264 N for Group C. The failure load of Group C was significantly higher than that of Group A and Group B (Figure 2D).

## 4. Discussion

Metaphyseal comminuted distal femoral fractures occur mainly in young patients with high-energy trauma and in older patients with low-energy trauma [3,4]. Non-surgical treatment requires long-term bed rest, which can easily lead to complications such as pressure ulcers, hypostatic pneumonia and deep vein thrombosis of the lower extremities. Therefore, it is only suitable for a few patients, such as those with a stable fracture, no obvious displacement of the fracture, or elderly patients who are already on long-term bed rest due to poor underlying health conditions [19]. At present, for the metaphyseal comminuted distal femoral fractures, most scholars advocate early surgical treatment, which is aimed at early functional exercise, preventing knee joint stiffness, and reducing a series of complications caused by long-term bed rest [20]. Traditional treatment methods for distal femoral fractures include angle-blade plates, dynamic condylar screws, intramedullary nails, etc. However, these internal fixators revealed their own disadvantages during the process of use, such as re-displacement, malunion, nonunion, etc. These defects limited the application of traditional internal fixators in the treatment of complex distal femoral fractures [21]. In recent years, the use of locking plates has significantly improved the surgical efficacy of distal femoral fractures [22]. The locking plate has the advantages of minimally invasive fixation and angular stability, which makes up for the shortage of previous internal fixators. A locking plate does not have the compression effect of a traditional plate on the surface of the bone cortex and preserves the necessary periosteum blood supply for fracture healing. Moreover, the screw is locked with the plate, similar to the built-in external fixation scaffold, which can obtain a relatively stable and reliable fixation. For femoral condylar fractures with severe osteoporosis or multi-plane intra-articular fracture fragments, multiple angle-stabilized locking screws provide a better anchoring force than conventional cancellous screws [23]. However, with the increasing application of locking plates, complications such as delayed union, malunion, nonunion and internal fixation failure have gradually increased. The incidence of nonunion for distal femoral fractures treated with lateral locking plates is reported to be 0–10%, which may be related to the insufficient stability of a single-plate fixation and the fracture type [7,8,9,24,25,26]. Clinically, the locking plate is placed on the lateral side of the distal femur based on the principle of a tension band: there are tension sides (lateral) and pressure sides (medial) of the femur under load. In order to convert the tensile load, which is not beneficial to fracture healing, into compressive load, the locking plate should be placed on the lateral side. However, the use of this principle must meet a basic condition, that is, the medial cortex (pressure side) must be intact to provide adequate support. When the medial cortex is shattered and does not provide adequate support, the lateral plate may not provide a stable fixation for this fracture type. The lateral locking plate alone was used to fix the metaphyseal fracture of the distal femur. Due to the lack of adequate medial support, flexion deformity occurs when the fracture is subjected to axial stress, resulting in delayed healing, nonunion or internal fixation failure [27,28,29,30]. For this reason, combined fixation, with an additional medial plate or a retrograde femoral intramedullary nail, is often used clinically [14,31]. For comminuted distal femoral fractures lacking medial bone support, a medial locking plate is added to the lateral LISS plate to provide adequate support, which meets the prerequisite for the use of the tension band principle.

Biomechanical experiments can test the stability in fracture surgery of different internal fixation constructs to guide clinical application [32,33,34]. Our results showed that the dual-plate group had a higher anti-axial and anti-torsion ability than the retrograde femoral nail combined with the LISS group (RFN + LISS construct). The torsion resistance of locking plate was stronger than that of the intramedullary nail, as has been confirmed by many studies [35]. However, biomechanical studies have demonstrated that the axial stress resistance of the intramedullary nail is stronger than a plate in distal femoral fractures [36]. This is contrary to the conclusion of our study, which may be caused by the different lengths of screws used at the proximal end of the model between the lateral plates of two of the groups. In the RFN + LISS group, only a single cortical screw was used at the proximal end of the LISS due to the block of the proximal intramedullary nail, which may be an important factor affecting our experimental results. At the same time, another similar cadaver and artificial bone biomechanical experiment reached the same conclusion as ours, and the dual-plate group had stronger axial and torsional stress resistance than the RNF + LISS group [17]. These results indicate that the overall stability of fracture fixation can be improved by increasing the strength of medial support compared with the axial central stability of the femur.

Ours is different from other studies; we used a tibial retrograde nail instead of a medial plate for the first time to restore the medial stability of the distal femoral fractures because the placement of the medial plate may damage the bone blood at the fracture site and thus affect the healing of the fracture [16,26]. At the same time, due to the lack of a special medial femoral plate, this may lead to loss of femoral reduction [37,38]. According to the results of our recent clinical studies, the use of a retrograde tibial nail to restore the medial stability of the distal femoral fracture may be a better internal fixation [39,40]. In order to test the biomechanical stability of the different internal fixations, we compared it with the femoral retrograde nail combined with the LISS and the dual plate. This study showed that the RTN + LISS group for metaphyseal comminated distal femoral fractures was significantly superior to the dual-plate group and the RFN + LISS group in terms of axial stiffness and torsion stiffness, indicating that the addition of medial tibial retrograde intramedullary nails could have made the internal fixation system more stable and significantly reduce fretting at the fracture end. However, a concern is that for comminuted fractures, too high a fixation strength may not be conducive to fracture healing. Fixation stability that matches the severity of the fracture is a prerequisite for fracture healing; the appropriate mechanical stimulation can promote bone remodeling and bone healing under the appropriate fixation strength [41,42]. However, too high a fixation strength and stiffness may result in too stable and insufficient mechanical stimulation to induce callus formation to promote the secondary healing of fractures [43]. Whether this internal fixation method can achieve better clinical healing still needs further clinical controlled studies to confirm this.

This study has the following limitations. First, the in vitro mechanical experiment cannot completely simulate the complex mechanical environment of the human body, so the conclusion is only valuable for reference. This study only analyzes axial and torsional loads, which are bound to be different from the mechanical conduction mode of the knee joint during human activities. Secondly, this experiment did not compare the effects of different plate lengths and screw number on the biomechanics at the fracture site. Therefore, although the results of this study showed that the retrograde tibial nail combined with an LISS had stronger biomechanical strength than the dual-plate group and the retrograde femoral nail combined with an LISS group, there was still a lack of more biomechanical evidence, and further clinical trial support on whether better fixation strength and fracture healing could be obtained clinically.

## 5. Conclusions

The treatment of comminuted distal femoral fractures with an RTN combined with an LISS construct has higher biomechanical fixation strength than that of a retrograde femoral nail combined with an LISS and double-plate constructs. The RTN combined with an LISS construct may be a good internal fixation combination for the treatment of comminuted distal femoral fractures but still needs to be validated by further clinical studies.

## Data Availability

The data presented in this study are available from the corresponding author.
